# Impacts of DRG point-based payment system on healthcare resource utilization and provider behavior: a pilot quasi-experimental study in China

**DOI:** 10.3389/fpubh.2025.1678259

**Published:** 2025-10-07

**Authors:** Xiao Huang, Jinyun Zhu, Jinghua Zhang

**Affiliations:** ^1^School of Business, Macau University of Science and Technology, Macau, Macau SAR, China; ^2^School of Humanities and Management, Southwest Medical University, Luzhou, Sichuan, China; ^3^Ganzhou Municipal Health Commission in Jiangxi Province of China, Ganzhou, Jiangxi, China; ^4^Institute of Development Economics, Macau University of Science and Technology, Macau, Macau SAR, China

**Keywords:** diagnosis-related group payment, diagnosis-related group point-based payment, hospitalization cost, length of stay, patient selection, premature discharge

## Abstract

**Background:**

Developing countries commonly face challenges regarding budget constraints and inadequate cost-accounting capabilities during the implementation of a Diagnosis-Related Group (DRG) payment system. China has initiated pilot reforms of the Diagnosis-Related Group point-based payment system (DRG-PBPS) in 40 cities. DRG-PBPS, using historical cost data, integrates global budgeting with case-based point-weighted payments. In this study, its impact on inpatient resource utilization is evaluated and potential strategic behaviors of healthcare providers are examined.

**Methods:**

Using administrative data from 15,744 inpatient records of cerebral infarction cases from January 2018 to December 2022 in a Chinese city, this study uses a difference-in-differences (DID) approach to evaluate the effects of the DRG-PBPS reform on hospitalization costs and length of stay. Both the changes in the Charlson Comorbidity Index (CCI) score and 14-day, 30-day, and 90-day readmission rates between the reform and control groups were compared before and after implementation to assess whether providers were involved in patient selection and premature discharge.

**Results:**

After DRG-PBPS implementation, hospitalization costs decreased by 9.7% (*p* < 0.01), and length of stay decreased by 6.5% (*p* < 0.05). No significant changes were observed in CCI or 14-day and 90-day readmission rates, whereas 30-day readmissions fell by 2.0% (*p* < 0.05). These findings were robust across multiple sensitivity analyses, and the estimated effects of DRG-PBPS were broadly consistent across hospitals of different levels.

**Conclusion:**

The implementation of DRG-PBPS significantly reduced inpatient resource utilization without inducing adverse provider behavior. China’s pilot practice illustrates that the DRG-PBPS serves as an effective alternative to the fee-for-service model. For developing countries with constrained budgets and underdeveloped cost-accounting systems, DRG-PBPS provides a feasible strategy for adopting DRG-based payment systems in inpatient care.

## 1 Introduction

### 1.1 Background

The increase in healthcare expenditures has led to the widespread adoption of Diagnosis-Related Group (DRG)-based payment systems as the predominant model for hospital reimbursement in many countries over the past decade (1–4). DRG-based reimbursement mechanisms are designed to transfer financial responsibility for marginal treatment costs to hospitals, thereby incentivizing cost control and reducing overall healthcare spending (5–8). In recent years, the DRG payment system has gained increasing traction in developing countries (9–11). However, these countries face unique challenges in localizing DRG systems because of financial constraints and distinct institutional frameworks (12). Effective implementation necessitates adaptation of core DRG components—such as classification logic, relative weight calibration, and payment parameters—to align with the specific characteristics of local healthcare systems (13). These adaptations can substantially alter provider incentives, which carries potential implications for both healthcare expenditures and quality of care (14, 15).

Since 2020, China has piloted a Diagnosis-Related Group Point-Based Payment System (DRG-PBPS) in 40 cities to meet stringent cost-control requirements (16). DRG-PBPS integrates case-based payments with a regional global budget (17). This design differs from traditional global budget models, in which expenditure caps are imposed at the hospital level (18), and from hybrid models used in Taiwan region and Germany, where point-based global budgets are combined with fee-for-service (FFS) reimbursement (19–22). Instead, DRG-PBPS establishes a regionally coordinated reimbursement framework that aligns case-based payments with overall budgetary control. These structural differences generate distinct incentive effects for providers. However, existing evidence is mixed: while Liao et al. (23) reported reductions in both per-patient costs and length of stay (LOS), another study on pediatric services found that LOS decreased but overall costs remained unchanged (17). Thus, the impact of DRG-PBPS on inpatient resource utilization remains uncertain.

At its core, DRG-PBPS is a stringent cost-control mechanism designed to prevent regional healthcare expenditures from exceeding preset budgetary limits (16, 17). Under this system, financial risk is fully transferred to providers, increasing the likelihood of strategic behavioral responses. International evidence shows that under DRG-based payment systems, hospitals frequently adopt strategies to safeguard their financial interests, including patient selection (favoring low-cost or low-risk cases), reduction of necessary services, premature discharge, and upcoding (24–27). While these behaviors may superficially appear to lower resource utilization, they distort the true impact of DRG reforms. Similar provider responses may emerge following DRG-PBPS implementation, yet these issues have not been directly examined. In China, the extensive use of expert review systems and big data–driven medical monitoring improves the detection of unnecessary service reductions and upcoding (28, 29). However, patient selection and premature discharge remain comparatively easy to implement, entail limited liability, and can still yield financial gains for providers (30–32). The adoption of such strategies significantly complicates the evaluation of DRG-PBPS effects on inpatient resource utilization, yet these concerns remain largely underexplored in the existing literature.

This study employs a quasi-natural experimental design leveraging the implementation of DRG-PBPS in the study city. In January 2020, the city shifted hospital reimbursement for secondary and tertiary hospitals under the Urban and Rural Resident Basic Medical Insurance (URBMI) scheme from FFS to DRG-PBPS. Crucially, the reform applied only to local URBMI enrollees, while nonlocal residents continued to receive care under FFS. This institutional arrangement creates a natural reform and control group, providing a valuable opportunity to evaluate the impact of DRG-PBPS on inpatient resource utilization and to assess potential strategic responses by healthcare providers.

Cerebral infarction is an appropriate condition for assessing the impact of the DRG-PBPS on inpatient healthcare resource utilization and the strategic responses of healthcare providers. It is highly prevalent in the general population and demonstrates substantial variation in clinical severity (33, 34). The acute onset of cerebral infarction minimizes patient self-selection bias, as individuals have little discretion to delay hospitalization (35, 36). Immediate intervention is essential, ideally within 4.5 h, to prevent irreversible brain tissue damage (35). Accordingly, admission patterns for cerebral infarction are far less affected by external shocks, such as the COVID-19 pandemic, than conditions that permit elective or delayed admission. Treatment options range from conservative drug therapy to intravenous thrombolysis and endovascular recanalization (35–37), resulting in wide differences in resource consumption. Consequently, these characteristics make cerebral infarction a suitable condition for quasi-experimental evaluation of DRG-PBPS–driven cost control and potential provider behavioral responses.

This study evaluates the impact of DRG-PBPS on inpatient healthcare resource utilization and examines whether the reform induces strategic provider behaviors, specifically patient selection and premature discharge. By analyzing both resource utilization and provider behavior outcomes, it contributes new empirical evidence to the literature on hospital payment reforms in resource-constrained settings. The findings provide insights into the effectiveness of DRG-PBPS as well as its potential unintended consequences, offering valuable policy implications for countries undertaking similar DRG-based payment reforms.

### 1.2 Institutional design and theoretical framework

#### 1.2.1 The design of the DRG-PBPS in the studied city

The studied city, located in central China and serving as a provincial capital, has both a population size and an economic development level that place it in the middle range nationally. In January 2020, the city implemented the DRG-PBPS reform within its URBMI scheme. It was among the first cities in China to introduce the DRG-PBPS. The DRG-PBPS covers more than 90% of inpatient costs in hospitals. However, costs related to mental illnesses and certain cancers are excluded from DRG-PBPS coverage. The DRG-PBPS reform is a purely supply-side intervention, with no changes to copayment rates, deductibles, or benefit packages on the demand side.

Based on the conceptual model of the DRG payment system proposed by Mathauer and Wittenbecher (12), we developed a schematic diagram illustrating the operational process of DRG-PBPS in the study city (Appendix Figure A1). In implementing DRG-PBPS in the study city, patient grouping is primarily based on the China Healthcare System DRG (CHS-DRG) framework, with modifications introduced to reflect local characteristics. Specifically, patients are classified into diagnosis-related groups according to primary diagnosis codes (International Classification of Diseases, Tenth Edition, ICD-10), procedure codes (International Classification of Diseases, Ninth Revision, Clinical Modification, ICD-9-CM-3), the presence of comorbidities or complications, as well as individual characteristics. In 2020, a total of 693 diagnosis-related groups were formally established.

Under the DRG-PBPS, each DRG category is allocated a fixed number of points annually, and hospitals receive corresponding points for each inpatients admitted. Fixed points are derived from 3 years of historical cost data using a designated reference group. Specifically, the study city designated “Gallbladder Calculi with Chronic Cholecystitis” as the reference group, which was standardized at 1,000 points. Points for other DRG categories are determined by multiplying the ratio of their average cost to the reference group’s cost by 1,000.

As shown in Formula 1, the point value is calculated by dividing the URBMI regional budget allocated for DRG-PBPS by the cumulative points of all hospitals in the region, thereby ensuring that total expenditures remain within predetermined budgetary limits (17, 38, 39). Therefore, the DRG-PBPS uses a floating-point value that varies annually and is determined only at the end of the year, as the total points across all hospitals can be calculated only at that time. This innovative design demonstrates that hospital reimbursement amounts are determined by three key factors: regional budget, each hospital’s service volume (total accumulated points), and the aggregate service activity volume (total points) across all hospitals within the region.

A hospital’s total reimbursement amount is calculated by multiplying its total accumulated points by the point value (Formula 2). Beyond this base formula, the study city incorporates adjustment factors to further adjust reimbursement levels according to institutional characteristics, including hospital grade, case-mix index (CMI), the proportion of patients over 65, and priority specialty services. This framework enhances budget predictability while promoting equitable compensation, thereby controlling total expenditures within fiscal constraints and mitigating the risks of deficits and cost inflation.


(1) $$\text{Point value}=\frac{\begin{array}{l} \text{The total regional budget of URBMI}\;\\ \text{funds available for}\;\mathrm{DRG}-\text{PBPS} \end{array}}{\sum (\begin{array}{l} \text{Fixed points assigned to}\;a\;\mathrm{DRG}\;\text{group}\\ \times \text{number of inpatient cases in that}\;\mathrm{DRG}\;\text{group} \end{array})}\;$$

(2) $$\begin{array}{l} \text{Reimbursement}=\text{Total points of the hospital}\times \text{Point value}\\ \times \text{Adjustment factors}\; \end{array}$$

#### 1.2.2 Theoretical foundation

This section employs principal–agent theory alongside the institutional features of the DRG-PBPS to construct the theoretical framework underpinning our analysis. In health economics, the payer–provider relationship is often conceptualized as a principal–agent problem (40, 41): insurers (principals) seek cost control and efficiency, while providers (agents), who possess superior clinical information, may exploit this asymmetry to maximize revenue (24, 42–44). Under FFS, this asymmetry fosters moral hazard, as hospitals increase service volume and intensity (45–47). To counteract such distortions, DRG standardize case-based payments and shift provider incentives toward efficiency (48). To counteract such distortions, DRG payment systems standardize case-based reimbursements, thereby redirecting provider incentives toward efficiency (1, 3, 49). Within this framework, the DRG-PBPS operates as a performance-based contract designed to align provider behavior with the principal’s objectives (16, 24). Nonetheless, providers remain imperfect agents who may exploit informational advantages through strategies such as patient selection and premature discharge to offset income losses (25, 27). Accordingly, the structure of the payment system is pivotal in determining incentive compatibility and shaping the magnitude of unintended consequences (15, 20, 21, 50, 51).

The central innovation of the DRG-PBPS lies in its floating point value mechanism combined with regional budget coordination. Unlike fixed case-based DRG payments, the monetary value of each point is retrospectively determined at the end of the fiscal year by dividing the regional budget by the total points accumulated across all hospitals. This retrospective calculation creates a dynamic adjustment process: if hospitals collectively increase service volumes, the reimbursement value of each point declines (52, 53), thereby diluting individual financial gains and raising the effective cost per inpatient episode or day of stay. From an economic perspective, this design enhances incentive compatibility because opportunistic increases in service intensity yield diminishing marginal returns (54, 55). Consequently, hospitals are encouraged to deliver care efficiently rather than maximize revenue through excessive treatment, which restrains unnecessary costs and prevents unwarranted extensions of hospitalization.

Beyond mitigating moral hazard, the DRG-PBPS also functions as a risk-sharing contract among hospitals that further stabilizes average hospitalization costs across the region. Because reimbursement depends not only on an individual hospital’s activity but also on the collective behavior of all hospitals, the system embeds providers in a quasi-cooperative environment (17, 20, 21, 56). Over-provision by one hospital reduces the point value for all, thereby creating implicit peer-monitoring incentives and fostering collective fiscal discipline This arrangement reflects a model of regional budget coordination, whereby hospitals collectively share responsibility for adhering to expenditure caps (21, 52). Although hospitals within a region could theoretically collude to inflate costs, such arrangements are unstable: hospitals that abstain from collusion retain surpluses under the year’s standard, while those that participate incur losses, leading to the eventual collapse of collusion under individual rationality (20, 57).

Finally, the DRG-PBPS incorporates adjustment mechanisms to mitigate perverse incentives for patient selection. Adjustment factors—including hospital grade coefficients, CMI weights, and demographic allowances for the proportion of patients over 65—compensate for greater clinical complexity, thereby ensuring that higher-cost or longer-stay patients are not systematically excluded (39). These measures reduce the risk of adverse selection against severely ill patients, a frequent unintended consequence of DRG payment systems.

In sum, the design of the DRG-PBPS embodies the logic of principal–agent theory: it addresses information asymmetry and moral hazard through case-based payments and the floating point-value mechanism, enforces fiscal discipline via regional budget coordination, and balances efficiency with equity through risk adjustment.

#### 1.2.3 Expected effects on healthcare resource utilization and provider behavior

The DRG-PBPS is a case-based payment in which reimbursement is determined by disease category and severity, irrespective of the actual cost of services (49). Because hospitals bear the financial risk (6), prolonging LOS or increasing service volumes does not raise revenue but instead escalates costs. Furthermore, within the DRG-PBPS design, the floating point-value mechanism, together with the system’s risk-sharing arrangement among hospitals, discourages hospitalization extensions and excessive treatment intensity. Accordingly, we expect that the introduction of the DRG-PBPS will lead to reductions in both hospitalization costs and LOS.

Patient selection is one of the simplest strategic responses under DRG-PBPS (58). Its core principle of “same DRG group, same payment standard” links provider profits inversely to patient resource consumption: hospitals profit when treatment costs fall below the standard and incur losses when they exceed it. Evidence from other DRG systems indicates that such incentives can lead to selection based on age, ethnicity, or comorbidities (59, 60). under the DRG-PBPS design, age is incorporated into grouping weights and ethnicity is uniformly managed, reducing the likelihood of selection on these grounds. Complications, however, remain a key determinant of treatment costs. Because point values are calculated from historical averages, they often underestimate the costs of complex cases, creating the familiar dynamic of “profiting from mild cases and incurring losses on severe cases” (30). This dynamic incentivizes hospitals to favor patients with controllable costs while avoiding those with multiple comorbidities (31). To mitigate these risks, the DRG-PBPS applies adjustment mechanisms such as the CMI, which partially compensates for clinical complexity. Whether patient selection emerges in practice depends on the balance between cost-control pressures and the effectiveness of compensation mechanisms.

Under the DRG-PBPS, premature discharge is incentivized because it reduces per-case costs while allowing hospitals to increase admissions and, consequently, reimbursement. However, premature discharge may compromise care quality, resulting in higher readmission rates, deterioration of the acute condition, and even mortality (61–66). Although strong cost-control pressures could encourage this behavior (49, 61), the DRG-PBPS incorporates safeguards to mitigate such risks. Hospitals continue to compete for patients, and quality remains a key determinant of patient choice (4). Specialized providers may receive supplementary reimbursement coefficients, while insurers closely monitor 30-day readmission rates as indicators of care quality. Accordingly, although the DRG-PBPS reinforces incentives to shorten LOS, competition, regulatory oversight, and reputational considerations are expected to limit premature discharge, compelling hospitals to balance cost-containment objectives with the preservation of care quality.

## 2 Materials and methods

### 2.1 Data source and sampling

The data for this study were sourced from the provincial comprehensive hospital management evaluation platform database, which records all inpatient medical records and serves as the regulatory foundation for the local health administrative departments, thus ensuring both the reliability and authenticity of the data. The data inclusion criteria were as follows:

Disease classification: Cerebral infarction cases were identified based on the primary diagnosis code I63 from the ICD-10.

LOS: Patients with a LOS between 1 and 59 days were included in the study, as this range falls within the scope of the DRG-PBPS. Cases with a LOS of less than 1 day or more than 60 days were excluded, as they are reimbursed through alternative payment methods.

Insurance type: Inclusion was limited to patients enrolled in the URBMI, encompassing both local and non-local residents. Patients covered under other insurance schemes were excluded, as these groups were not affected by the DRG-PBPS reform.

Hospital certification: Only secondary and tertiary general hospitals were selected, as the DRG-PBPS reform was specifically applied to these hospitals. In the Chinese hospital grading system, secondary hospitals provide regional health services with at least 100 inpatient beds, while tertiary hospitals are large, comprehensive institutions that provide advanced referral services and engage in teaching and research (67). Traditional Chinese medicine hospitals, which use separate disease-specific pricing under URBMI, were excluded to avoid policy interference.

Study period: The data collection period spanned from January 2018 to December 2022, covering 2 years before to 3 years after the reform.

Ultimately, the sample includes 15,744 inpatient records of cerebral infarction cases. The sample was drawn from 10 hospitals, comprising five secondary hospitals and five tertiary hospitals, all of which were public institutions. Private hospitals were excluded due to incomplete medical records. The dataset contains patient and hospital characteristics, clinical diagnoses and treatment measures, admission and discharge information, resource utilization at discharge, and policy-related variables. To maintain patient confidentiality, all personal identifiers were anonymized prior to analysis.

### 2.2 Statistical methods

A difference-in-differences (DID) model was employed to evaluate the impact of the DRG-PBPS. The reform specifically targeted URBMI-insured local residents, who constituted the reform group, whereas nonlocal URBMI-insured residents served as the control group. The DID analysis used January 1, 2020, as the policy implementation cutoff point. Hospitalization costs per case and LOS served as the primary indicators of healthcare resource utilization.

To investigate the drivers of changes in total hospitalization costs, we conducted a mechanism analysis by disaggregating inpatient costs into three categories: drugs/consumables, services/treatments, and diagnostics (20, 39). Drug/consumable costs include prescribed medications, disposable medical materials, and therapeutic supplies. Service/treatment costs comprise physician fees, nursing care, procedures, and ward-related charges (excluding drugs and diagnostics). Diagnostic costs cover laboratory tests, imaging, and other diagnostic examinations. We then applied the same DID model used in the main analysis to estimate the reform’s impact on each cost component separately.

All costs were originally recorded in Chinese yuan (CNY) and were converted to US dollars (USD) using the World Bank’s annual average exchange rate for 2022 (1 USD = 6.73 CNY) to ensure consistency and international comparability (68).

To explore whether the DRG-PBPS reform induced patient selection behaviors among healthcare providers, changes in the Charlson Comorbidity Index (CCI) between the reform and control groups were compared before and after the reform. The CCI, which is calculated on the basis of secondary diagnoses, is used to measure patient severity, with higher scores indicating greater clinical complexity (69–71). In a DID context, unchanged or increased CCI scores in the reform group as relative to those in the control group would suggest that providers had not engaged in selective admission practices (72).

Premature discharge practices often reflect inadequate treatment and are associated with increased hospital readmissions (61–66). To evaluate the potential for premature discharge under the DRG-PBPS, we examined unplanned readmission rates within 14, 30, and 90 days following discharge among patients with the same primary diagnosis of cerebral infarction (ICD-10 code I63) (61–63, 73). An increase in readmission rates within these intervals would provide evidence of systematic premature discharge practices.

Control variables included age, gender (male, female), marital status (married, unmarried), and emergency admission status (emergency, non-emergency). Additional variables accounted for principal diagnoses (dummy variable of the first six digits of ICD-10 codes), the presence of comorbidities (binary variable), and the number of secondary diagnoses. Procedure-related variables included whether the patient received any procedure (binary variable) and whether the procedure was classified as complicated, defined as a grade 3 or grade 4 surgery (binary variable) (39). In this study, the term procedure is defined according to the ICD-9-CM-3 standards, covering all invasive diagnostic and therapeutic interventions performed during hospitalization.

The variable tertiary_hospital in the heterogeneity analysis is a binary indicator, coded as 1 for tertiary hospitals and 0 for secondary hospitals.

### 2.3 Empirical models

We formulated a DID model to identify the impact of DRG-PBPS implementation. For patient i in hospital h at time t, we employed the following equation:


(3) $$Y_{\mathrm{ith}}=\alpha +{\text{δTreat}}_{i}+{\text{βPost}}_{t}\times {\text{Treat}}_{i}+\gamma X_{\mathrm{ith}}+H_{h}+\tau_{t}+\varepsilon_{\mathrm{ith}}\;$$

where $Y_{\mathrm{ith}}$ denotes outcome variables, which include costs, LOS, CCI, and readmission rates within 14,30 and 90 days. Costs and LOS were LN-transformed due to skewness in their distributions. ${\text{Treat}}_{i}\;$ is a dummy that is equal to one for patients in the local URBMI group (reform group) and zero for patients in the nonlocal URBMI group (control group). ${\text{Post}}_{t}$ is a dummy that is equal to one for discharges occurring after January 1, 2020. The interaction term coefficient (*β*) captures the policy effect.

$X_{\mathrm{ith}}$ represents patient-level characteristics, including age, gender, marital status, emergency admission status, principal diagnoses, comorbidities, the number of secondary diagnoses, receiving procedures and receiving complicated procedures. $H_{h}$ is a hospital fixed-effect vector, and $\tau_{t}$ is a year–month fixed-effect vector. Since the year-month fixed effect was added to the model, the coefficient of $\;{\text{Post}}_{t}$ was not estimated separately. Standard errors ( $\varepsilon_{\mathrm{ith}}$ ) were clustered at the hospital-month level.

All the statistical analyses were performed using Stata version 17.0 (StataCorp LLC, College Station, TX, United States). We used 5% as the significance level.

## 3 Results

### 3.1 Descriptive statistics

Table 1 presents the descriptive statistics for the analytical cohort. Among the 15,744 patients included, the average age was69.77 years, and 58.13% were male, 90.63% were married, 8.97% were admitted via emergency departments, and 20.03% presented with comorbidities. The average number of secondary diagnoses was 4.63. Following the reform, hospitalization costs rose slightly in the control group (from 2,221.75 to 2,320.16 USD), whereas costs in the reform group remained essentially stable (from 1719.90 to 1725.47 USD). The decline in LOS was more pronounced in the reform group (from 10.51 to 10.19 days) compared with the control group (from 11.14 to 10.99 days). Regarding the CCI, the reform group experienced a smaller increase (from 2.04 to 2.32) than the control group (from 2.12 to 2.45). Additionally, the 30-day readmission rate declined in the reform group (from 4.55 to 3.44%) but slightly increased in the control group (from 5.09 to 5.18%). All the variables analyzed presented means and standard deviations that fell within the expected ranges, with no significant statistical outliers detected.

**Table 1:** Descriptive statistics of cerebral infarction inpatients in city A (2018–2022).

Panel A: descriptive statistics for categorical variables									
Variables	Full sample *N* (%) (1)	Pre-reform		$\chi^{2}$	*p* value	Post-reform		$\chi^{2}$	*p* value
		Local *n* (%) (2)	Nonlocal *n* (%) (3)			Local *n* (%) (4)	Nonlocal *n* (%) (5)		
Gender									
Male	9,152 (58.13)	2,958 (55.90)	584 (64.67)	24.272	<0.001	3,910 (56.77)	1700 (63.86)	39.802	<0.001
Female	6,592 (41.87)	2,334 (44.10)	319 (35.33)			2,977 (43.23)	962 (36.14)		
Marital status									
Married	14,268 (90.63)	4,879 (92.20)	812 (89.92)	5.333	0.021	6,238 (90.58)	2,339 (87.87)	15.423	<0.001
Unmarried	1,476 (9.38)	413 (7.80)	91 (10.08)			649 (9.42)	323 (12.13)		
Emergency admission									
Yes	1,413 (8.97)	194 (3.67)	61 (6.76)	18.653	<0.001	667 (9.68)	491 (18.44)	138.251	<0.001
No	14,331 (91.03)	5,098 (96.33)	842 (93.24)			6,220 (90.32)	2,171 (81.56)		
Comorbidities									
Yes	3,154 (20.03)	727 (13.74)	153 (16.94)	6.504	0.011	1,523 (22.11)	751 (28.21)	39.346	<0.001
No	12,590 (79.97)	4,565 (86.26)	750 (83.06)			5,364 (77.89)	1911 (71.79)		
Receiving procedures									
Yes	621 (3.94)	170 (3.21)	86 (9.52)	77.562	<0.001	205 (2.98)	160 (6.01)	48.069	<0.001
No	15,123 (96.06)	5,122 (96.79)	817 (90.48)			6,682 (97.02)	2,502 (93.99)		
Receiving complicated procedures									
Yes	479 (3.04)	135 (2.55)	72 (7.97)	70.223	<0.001	145 (2.11)	127 (4.77)	49.289	<0.001
No	15,265 (96.96)	5,157 (97.45)	831 (92.03)			6,742 (97.89)	2,535 (95.23)		
14-day readmission									
Yes	198 (1.26)	81 (1.53)	15 (1.66)	0.086	0.769	69 (1.00)	33 (1.24)	1.027	0.311
No	15,546 (98.74)	5,211 (98.47)	888 (98.34)			6,818 (99.00)	2,629 (98.76)		
30-day readmission									
Yes	662 (4.20)	241 (4.55)	46 (5.09)	0.509	0.475	237 (3.44)	138 (5.18)	15.456	<0.001
No	15,082 (95.80)	5,051 (95.45)	857 (94.91)			6,650 (96.56)	2,524 (94.82)		
90-day readmission									
Yes	1,236 (7.85)	465 (8.79)	76 (8.42)	0.132	0.716	478 (6.94)	217 (8.15)	4.173	0.041
No	14,508 (92.15)	4,827 (91.21)	827 (91.58)			6,409 (93.06)	2,445 (91.85)		
Tertiary_hospital									
Yes	6,177 (39.23)	1997 (37.74)	437 (48.39)	36.7349	<0.001	2,288 (33.22)	1,455 (54.66)	370.1624	<0.001
No	9,567 (60.77)	3,295 (62.26)	466 (51.61)			4,599 (66.78)	1,207 (45.34)		

Panel B: descriptive statistics of continuous variables									
Variables	Full sample Mean (S. D.) (1)	Pre-reform		*t*	*p* value	Post-reform		*t*	*p* value
		Local Mean (S. D.) (2)	Nonlocal Mean (S. D.) (3)			Local Mean (S. D.) (4)	Nonlocal Mean (S. D.) (5)		
Age (years)	69.77 (11.20)	70.11 (11.14)	66.39 (11.48)	−9.237	<0.001	70.52 (10.88)	68.28 (11.70)	−8.819	<0.001
Number of secondary diagnoses	4.63 (2.39)	3.89 (2.31)	4.66 (2.36)	9.224	<0.001	4.86 (2.32)	5.49 (2.33)	11.994	<0.001
hospitalization costs per case (USD)	1852.61 (2133.18)	1719.90 (1439.84)	2221.75 (2459.41)	8.559	<0.001	1725.47 (2011.73)	2320.16 (3151.86)	10.926	<0.001
Drug/consumable costs (USD)	952.18 (1371.93)	888.37 (869.10)	1111.30 (1476.16)	6.310	<0.001	879.62 (1258.87)	1212.75 (2166.97)	9.321	<0.001
Service /treatment costs (USD)	316.87 (645.36)	267.10 (416.59)	467.21 (859.54)	10.987	<0.001	293.86 (654.40)	424.35 (857.21)	7.977	<0.001
Diagnostic costs (USD)	550.42 (372.56)	514.47 (319.90)	608.74 (410.09)	7.826	<0.001	536.65 (353.93)	637.70 (474.02)	11.320	<0.001
Length of stay(days)	10.49 (5.84)	10.51 (5.49)	11.14 (6.67)	3.078	0.002	10.19 (5.70)	10.99 (6.50)	5.889	<0.001
Charlson comorbidity index	2.27 (1.29)	2.04 (1.15)	2.12 (1.15)	2.118	0.034	2.32 (1.35)	2.45 (1.39)	4.332	<0.001
Observations	15,744	5,292	903	–	–	6,887	2,662	–	–

The term “local” denotes residents enrolled in the URBMI, who constitute the reform group. “Nonlocal” refers to individuals in the control group. The “pre-reform” period ranges from 2018 to 2019, whereas the “post-reform” period covers the period of 2020 to 2022. Exchange rate: 1 USD = 6.73 CNY.

### 3.2 The impacts of the DRG-PBPS implementation on the utilization of healthcare resource

Table 2 presents the estimated effects of the DRG-PBPS payment on both hospitalization costs per case and LOS. Column (1) indicates that the DRG-PBPS payment reform significantly reduced hospitalization costs per case. Compared with those in the nonlocal group, the total hospitalization costs per case in the local group decreased by 9.7% (95% CI: −0.151 to −0.043, *p* < 0.01) following DRG-PBPS implementation. Column (2) shows that the DRG-PBPS payment reform reduced LOS by 6.5% (95% CI: −0.114 to −0.015, *p* < 0.05).

**Table 2:** Impacts of the DRG-PBPS implementation on hospitalization costs and length of stay.

Variables	LN (hospitalization costs per case)	LN (length of stay)
	(1)	(2)
${\text{Treat}}_{i}$	0.052^**^	0.033
	(0.007, 0.098)	(−0.011, 0.078)
${\text{Post}}_{t}\times {\text{Treat}}_{i}$	−0.097^***^	−0.065^**^
	(−0.151, −0.043)	(−0.114, −0.015)
Age (years)	0.001	−0.001^***^
	(−0.001, 0.001)	(−0.002, −0.001)
Gender(Male = 1)	0.001	−0.009
	(−0.015, 0.016)	(−0.024, 0.006)
Marital Status(Married = 1)	−0.057^***^	−0.060^***^
	(−0.085, −0.030)	(−0.089, −0.031)
Emergency admission (yes = 1)	0.103^***^	−0.014
	(0.065, 0.140)	(−0.047, 0.019)
Comorbidities(Yes = 1)	0.108^***^	0.054^***^
	(0.086, 0.131)	(0.032, 0.075)
Number of secondary diagnoses	0.047^***^	0.035^***^
	(0.042, 0.051)	(0.031, 0.039)
Receiving procedures (Yes = 1)	0.770^***^	0.139^**^
	(0.660,0.880)	(0.026,0.253)
Receiving complicated procedures (Yes = 1)	0.114^*^	0.016
	(−0.011, 0.240)	(−0.113, 0.144)
First 6 characters of ICD-10 code	Yes	Yes
Hospital fixed effect	Yes	Yes
Year-month fixed effect	Yes	Yes
Observations	15,744	15,744
*R* ^2^	0.420	0.137

***, **, and * indicate statistical significance at the 1, 5, and 10% levels, respectively. 95% confidence intervals s in brackets. Standard errors clustered at the hospital-month level. Since the year-month fixed effect was added to the model, the coefficient of $\;{\text{Post}}_{t}$ was not estimated separately. The ICD-10 refers to the International Classification of Diseases, 10th Revision. “Yes” indicates that the corresponding covariates were included in the regression model.

### 3.3 Mechanism analysis

To identify the drivers of the observed reductions in total hospitalization costs, a mechanism analysis was conducted by decomposing expenditures per admission into three major categories: drugs/consumables, services/treatments, and diagnostics. Using the same DID specification as in the main analysis (Equation 3), we estimated the reform’s impact on each component separately. Table 3 reports the results, with columns (1)–(3) presenting the effects of DRG-PBPS implementation on drugs/consumables, services/treatments, and diagnostic costs, respectively. Relative to the nonlocal group, the local group experienced an 11.9% reduction in drug/consumable costs (95% CI: −0.202 to −0.036, *p* < 0.01). By contrast, no significant effects were observed for service/treatment or diagnostic costs.

**Table 3:** Mechanism analysis of declining hospitalization costs.

Variables	Drug/consumable costs	Service/treatment costs	Diagnosis costs
	(1)	(2)	(3)
${\text{Treat}}_{i}$	0.075**	−0.035	0.057*
	(0.008, 0.141)	(−0.109, 0.040)	(−0.011, 0.126)
${\text{Post}}_{t}\times {\text{Treat}}_{i}$	−0.119***	−0.017	−0.007
	(−0.202, −0.036)	(−0.109, 0.075)	(−0.088, 0.073)
Age (years)	0.003***	0.002***	−0.002***
	(0.002, 0.004)	(0.001, 0.003)	(−0.003, −0.001)
Gender(Male = 1)	0.025**	−0.040***	−0.002
	(0.001, 0.049)	(−0.064, −0.015)	(−0.019, 0.015)
Marital Status(Married = 1)	−0.056***	−0.147***	−0.025
	(−0.095,-0.018)	(−0.194, −0.100)	(−0.055, 0.006)
Emergency admission (yes = 1)	0.159***	0.145***	0.048**
	(0.104, 0.213)	(0.085, 0.205)	(0.005, 0.092)
Comorbidities(Yes = 1)	0.135***	0.220***	0.025**
	(0.102, 0.168)	(0.184, 0.256)	(0.000, 0.051)
Number of secondary diagnoses	0.042***	0.074***	0.053***
	(0.035, 0.048)	(0.067, 0.080)	(0.048, 0.058)
Receiving procedures (Yes = 1)	0.799***	1.331***	0.762***
	(0.634, 0.964)	(1.159, 1.502)	(0.647, 0.876)
Receiving complicated procedures (Yes = 1)	0.410***	0.092	−0.332***
	(0.219, 0.601)	(−0.086, 0.270)	(−0.453, −0.211)
First 6 characters of ICD-10 code	Yes	Yes	Yes
Hospital fixed effect	Yes	Yes	Yes
Year-month fixed effect	Yes	Yes	Yes
Observations	15,744	15,744	15,744
*R* ^2^	0.289	0.436	0.417

***, **, and * indicate statistical significance at the 1, 5, and 10% levels, respectively. 95% confidence intervals s in brackets. Standard errors clustered at the hospital-month level. Since the year-month fixed effect was added to the model, the coefficient of $\;{\text{Post}}_{t}$ was not estimated separately. The ICD-10 refers to the International Classification of Diseases, 10th Revision. “Yes” indicates that the d:corresponding covariates were included in the regression model.

### 3.4 The impacts of the DRG-PBPS implementation on patient selection and premature discharge

The preceding analysis demonstrated that the implementation of DRG-PBPS significantly reduced hospitalization costs and length of stay in the reform group. To assess whether these reductions stemmed from strategic provider responses, the following section examines two common behaviors under prospective payment systems—patient selection and premature discharge—to determine their role in shaping the observed outcomes. A DID model was constructed using Equation 3 on the basis of CCI scores and readmission data to assess the potential occurrence of patient selection and premature discharges following DRG-PBPS implementation.

Table 4, Column (1), presents the changes in CCI scores following DRG-PBPS implementation. Compared with the nonlocal group, the local group exhibited no significant change in its CCI scores after reform ( β = − 0.072, 95% CI: −0.192 to 0.048, *p* > 0.1), suggesting the absence of patient selection on the basis of comorbidities.

**Table 4:** Impacts of the DRG-PBPS implementation on Charlson comorbidity index and readmission.

Variables	Charlson Comorbidity Index	14-day readmission	30-day readmission	90-day readmission
	(1)	(2)	(3)	(4)
${\text{Treat}}_{i}$	0.146***	0.001	0.011	0.017
	(0.046, 0.246)	(−0.009, 0.010)	(−0.007, 0.029)	(−0.005, 0.039)
${\text{Post}}_{t}\times {\text{Treat}}_{i}$	−0.072	−0.001	−0.020^**^	−0.018
	(−0.192, 0.048)	(−0.012, 0.010)	(−0.040,−0.001)	(−0.043, 0.008)
Age (years)	0.005***	−0.001	−0.001^*^	-0.001
	(0.003, 0.007)	(−0.001, 0.001)	(−0.001, 0.001)	(−0.001, 0.001)
Gender(Male = 1)	0.047**	−0.003	−0.005	−0.008^*^
	(0.008, 0.086)	(−0.007, 0.001)	(−0.011, 0.002)	(−0.017, 0.001)
Marital Status(Married = 1)	−0.049	0.003	−0.013^*^	−0.016^*^
	(−0.120, 0.022)	(−0.003, 0.009)	(−0.026, 0.001)	(−0.032, 0.001)
Emergency admission (yes = 1)	−0.023	0.003	−0.027^***^	−0.026^***^
	(−0.109, 0.063)	(−0.004, 0.010)	(−0.039, −0.015)	(−0.043,-0.009)
Comorbidities(Yes = 1)	–	0.005^*^	0.003	0.009
		(−0.000,0.010)	(−0.008, 0.015)	(−0.003, 0.020)
Number of secondary diagnoses	–	0.001	0.003^***^	0.006***
		(−0.000, 0.002)	(0.001, 0.005)	(0.004, 0.008)
Receiving procedures (Yes = 1)	–	−0.005	0.076^**^	0.005
		(−0.020, 0.010)	(0.005,0.148)	(−0.042,0.052)
Receiving complicated procedures (Yes = 1)	–	0.010	0.087^*^	0.013
		(−0.009, 0.028)	(−0.013, 0.187)	(−0.038, 0.063)
First 6 characters of ICD-10 code	Yes	Yes	Yes	Yes
Hospital fixed effect	Yes	Yes	Yes	Yes
Year-month fixed effect	Yes	Yes	Yes	Yes
Observations	15,744	15,744	15,744	15,744
*R* ^2^	0.114	0.012	0.058	0.044

***, **, and * indicate significance levels of 1, 5, and 10%, respectively. 95% confidence intervals s in brackets. Column (1) estimates the CCI based on Equation 3, excluding comorbidities,secondary diagnoses receiving procedures and receiving complicated procedures as covariates. Since the year-month fixed effect was added to the model, the coefficient of $\;{\text{Post}}_{t}$ was not estimated separately. The ICD-10 refers to the International Classification of Diseases, 10th Revision. “Yes” indicates that the listed covariates were included in the model. Standard errors clustered at the hospital-month level.

Table 4, columns (2)–(4), present the estimated effects of DRG-PBPS implementation on 14-day, 30-day, and 90-day readmission rates. The reform had no significant impact on 14-day and 90-day readmissions, while the 30-day readmission rate declined by 2.0% (95% CI: −0.040 to −0.001, *p* < 0.05). These findings suggest that DRG-PBPS implementation did not trigger premature discharge practices.

### 3.5 Parallel trend assumption test

**Figure 1:**
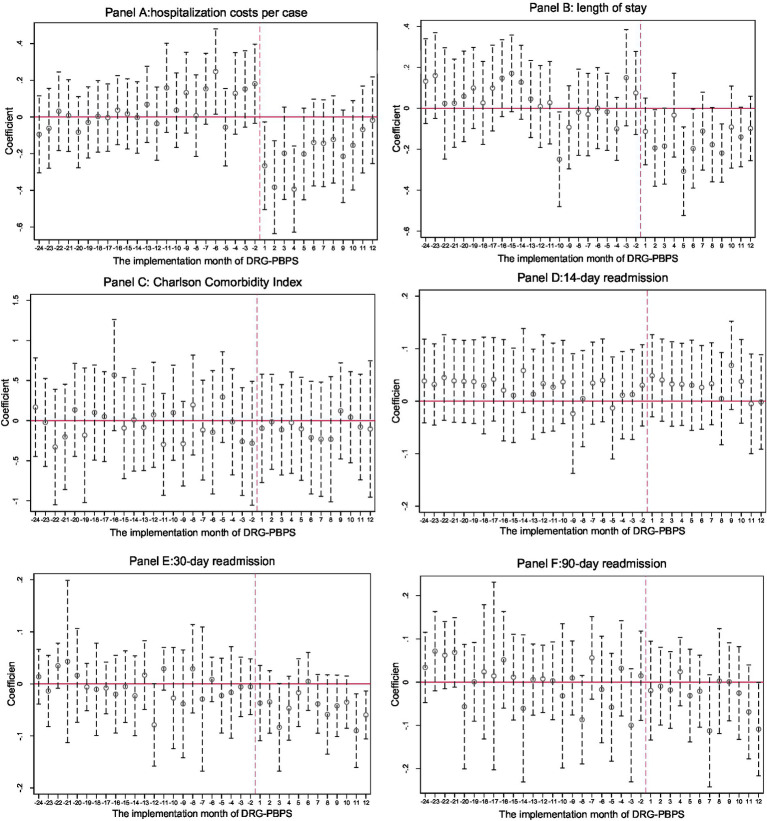
Parallel trend assumption test. These charts present the estimated coefficients of the interaction terms between the treatment group indicator and a set of time dummy variables representing the months following the implementation of the DRG-PBPS reform. Each vertical line denotes the 95% confidence interval, and standard errors are clustered at the hospital-month level. The month preceding the reform (December 2019) was excluded from the model to serve as the reference category, and its coefficient was set to zero. Estimates beyond the second year following reform implementation were trimmed to improve clarity.

A key assumption of the DID approach is that the outcomes of the reform and control groups follow parallel trends prior to implementation; otherwise, the validity of the estimates would be compromised. To examine this assumption, we employed an event study design. As shown in Figure 1, no statistically significant differences in pre-reform trends were observed between the reform and control groups for the CCI, 14-day readmission rate, 30-day readmission rate, and 90-day readmission rate. For hospitalization costs, the estimates were either statistically nonsignificant or exhibited inconsistent directions. For LOS, only one period showed a significant decrease before the reform, while the remaining periods were not statistically significant. These results suggest that no preexisting differences in outcomes existed between the groups prior to the implementation of policy reform, thereby supporting the internal validity of our findings.

### 3.6 Placebo test and robustness checks

To confirm that the estimated effects of the DRG-PBPS reform were not confounded by other factors, we conducted additional validity checks.

To rule out the possibility that the policy effects of the DRG-PBPS reform were influenced by other policies or random factors, we further conducted a policy placebo test. By artificially advancing the policy implementation date to 1 January 2019, i.e., by assuming that the implementation date of the DRG payment reform was 1 January 2019, the time point of the newly generated policy was fitted using DID. To isolate the true policy effect, the samples that were dated after the actual policy implementation date (1 January 2020) were excluded. As shown in Table 5, Column (1), none of the five results for the pseudo policy implementation pilot were significant.

**Table 5:** Placebo test and robustness checks.

Variables	Placebo test	PSM-DID	Exclude data during COVID-19
	(1)	(2)	(3)
Panel A: LN(hospitalization costs per case)			
${\text{Treat}}_{i}$	0.020	0.051^**^	0.118^***^
	(−0.027,0.067)	(0.005,0.097)	(0.050,0.185)
${\text{Post}}_{t}\times {\text{Treat}}_{i}$	−0.065	−0.096^***^	−0.106^**^
	(−0.244,0.114)	(−0.149,-0.042)	(−0.198,-0.014)
Hospital fixed effect	Yes	Yes	Yes
Year-month fixed effect	Yes	Yes	Yes
Control variables	Yes	Yes	Yes
Observations	6,195	15,725	5,602
Panel B: LN(length of stay)			
${\text{Treat}}_{i}$	0.029	0.030	0.041
	(0.025,0.084)	(−0.014,0.075)	(−0.016,0.097)
${\text{Post}}_{t}\times {\text{Treat}}_{i}$	0.004	−0.061^**^	−0.090^**^
	(0.078,0.086)	(−0.111,-0.011)	(−0.161,-0.019)
Hospital fixed effect	Yes	Yes	Yes
Year-month fixed effect	Yes	Yes	Yes
Control variables	Yes	Yes	Yes
Observations	6,195	15,725	5,602
Panel C: Charlson Comorbidity Index			
${\text{Treat}}_{i}$	0.185^**^	0.143^***^	0.158^**^
	(0.036,0.334)	(0.043,0.242)	(0.007,0.308)
${\text{Post}}_{t}\times {\text{Treat}}_{i}$	−0.083	−0.072	−0.044
	(−0.271,0.104)	(−0.191,0.047)	(−0.248,0.160)
Hospital fixed effect	Yes	Yes	Yes
Year-month fixed effect	Yes	Yes	Yes
Control variables	Yes	Yes	Yes
Observations	6,195	15,725	5,602
Panel D: 14-day readmission			
${\text{Treat}}_{i}$	0.007	0.001	0.007
	(−0.003,0.018)	(−0.009,0.010)	(−0.004,0.017)
${\text{Post}}_{t}\times {\text{Treat}}_{i}$	−0.014	−0.001	−0.006
	(−0.031,0.004)	(−0.011,0.010)	(−0.019,0.008)
Hospital fixed effect	Yes	Yes	Yes
Year-month fixed effect	Yes	Yes	Yes
Control variables	Yes	Yes	Yes
Observations	6,195	15,725	5,602
Panel E: 30-day readmission			
${\text{Treat}}_{i}$	0.016	0.011	0.030^**^
	(−0.004,0.036)	(−0.007,0.029)	(0.005,0.055)
${\text{Post}}_{t}\times {\text{Treat}}_{i}$	−0.025	−0.020^**^	−0.033^**^
	(−0.065,0.015)	(−0.040,-0.001)	(−0.063,-0.003)
Hospital fixed effect	Yes	Yes	Yes
Year-month fixed effect	Yes	Yes	Yes
Control variables	Yes	Yes	Yes
Observations	6,195	15,725	5,602
Panel F: 90-day readmission			
${\text{Treat}}_{i}$	0.029^*^	0.018	0.029^*^
	(−0.004,0.062)	(−0.004,0.040)	(−0.003,0.061)
${\text{Post}}_{t}\times {\text{Treat}}_{i}$	−0.021	−0.018	−0.021
	(−0.064,0.022)	(−0.043,0.007)	(−0.061,0.018)
Hospital fixed effect	Yes	Yes	Yes
Year-month fixed effect	Yes	Yes	Yes
Control variables	Yes	Yes	Yes
Observations	6,195	15,725	5,602

***, **, and * indicate statistical significance at the 1, 5, and 10% levels, respectively. 95% confidence intervals s in brackets. “Yes” indicates that the corresponding covariates were included in the model. Since the year-month fixed effect was added to the model, the coefficient of $\;{\text{Post}}_{t}$ was not estimated separately. The covariates used in Panel A and Panel B match those used in the first and second columns of Table 2, respectively. Panel C adopts the same covariates as those in the first column of Table 4. Panels D, E, and F use the covariates corresponding to the second, third, and fourth columns of Table 4, respectively. The estimated values of the covariates are reported in the Appendix Tables B1, B2, C3, C4, D1, D2. Standard errors are clustered at the hospital-month level.

We further evaluated robustness using a propensity score matching difference-in-differences (PSM-DID) approach. A 1:5 nearest-neighbor matching algorithm with a caliper of 0.2 was applied. Matching variables included age, gender, marital status, emergency admission, comorbidities, number of secondary diagnoses, and whether receiving procedures and receiving complicated procedures. After matching, the mean standardized difference of all covariates was below 5%, the pseudo R^2^declined from 0.045 to 0.002, indicating good covariate balance between the reform and control groups. Detailed diagnostics are presented in Appendix Tables C1, C2; Appendix Figures C1, C2. As shown in Table 5, Column (2), the PSM-DID estimates were consistent with the baseline DID results: hospitalization costs, LOS, and 30-day readmission rates declined significantly in the reform group, while CCI, 14-day readmission, and 90-day readmission rates showed no significant changes.

The COVID-19 pandemic overlapped with the study period and posed a potential source of confounding. To mitigate its impact on the results, data from January 2020 to April 2022—identified as the period most severely affected by the pandemic in the study cities—were excluded from the analysis (72). To ensure cohort comparability, the period from May to December 2019 was designated as the pre-reform baseline. As shown in Column (3) of Table 5, even after accounting for the potential confounding effects of COVID-19, the DRG-PBPS reform continued to significantly reduce hospitalization costs, LOS, and 30-day readmission rates. In contrast, the CCI,14-day readmission rate and 90-day readmission rate remained statistically unchanged. These results confirm that the primary findings were robust and not substantially biased by the pandemic period.

Overall, the placebo test, PSM-DID analysis, and adjustment for COVID-19 all confirm that the main findings are robust. The evidence supports the conclusion that the observed reductions in hospitalization costs, LOS, and 30-day readmissions can be attributed to the DRG-PBPS reform rather than to confounding influences or random variation.

### 3.7 Heterogeneity analysis across hospital levels

Hospitals of different levels may respond differently to the implementation of DRG-PBPS (52, 67). To assess this potential heterogeneity, we extend the baseline DID framework by incorporating hospital level into a Difference-in-Difference-in-Differences (DDD) model. Table 6 reports the estimation results, which indicate that, under the DDD specification, no significant differences are observed across hospital levels with respect to hospitalization costs, LOS, CCI and readmission rates. These findings suggest that the impact of DRG-PBPS is broadly consistent across hospitals of different levels.

**Table 6:** Heterogeneity analysis across hospital levels.

Variables	LN (hospitalization costs per case)	LN(length of stay)	Charlson Comorbidity Index	14-day readmission	30-day readmission	90-day readmission
	(1)	(2)	(3)	(4)	(5)	(6)
${\text{Treat}}_{i}$	0.075^**^	0.057^*^	−0.031	0.004	0.010	0.020
	(0.014,0.137)	(−0.001,0.115)	(−0.149,0.087)	(−0.006,0.015)	(−0.013,0.032)	(−0.009,0.048)
${\text{Post}}_{t}\times {\text{Treat}}_{i}$	−0.107^***^	−0.085^**^	0.071	−0.009	−0.017	−0.018
	(−0.180,-0.033)	(−0.150,-0.019)	(−0.075,0.217)	(−0.022,0.003)	(−0.043,0.009)	(−0.053,0.017)
${\text{Post}}_{t}\times$ *Tertiary_hospital*	0.075	0.051	0.453^***^	−0.012	0.016	0.016
	(−0.027,0.176)	(−0.041,0.144)	(0.246,0.661)	(−0.032,0.007)	(−0.020,0.052)	(−0.030,0.062)
${\text{Treat}}_{i}\times$ *Tertiary_hospital*	−0.065	−0.067	0.322^***^	−0.009	0.001	−0.008
	(−0.159,0.029)	(−0.156,0.023)	(0.135,0.508)	(−0.028,0.010)	(−0.036,0.039)	(−0.054,0.038)
${\text{Post}}_{t}\times {\text{Treat}}_{i}\times$ *Tertiary_hospital*	0.059	0.079	−0.221^*^	0.018	−0.002	0.008
	(−0.049,0.167)	(−0.022,0.180)	(−0.447,0.005)	(−0.004,0.040)	(−0.044,0.039)	(−0.044,0.061)
Hospital fixed effect	Yes	Yes	Yes	Yes	Yes	Yes
Year-month fixed effect	Yes	Yes	Yes	Yes	Yes	Yes
Control variables	Yes	Yes	Yes	Yes	Yes	Yes
Observations	15,744	15,744	15,744	15,744	15,744	15,744
*R* ^2^	0.423	0.140	0.117	0.013	0.059	0.044

***, **, and * indicate statistical significance at the 1, 5, and 10% levels, respectively. 95% confidence intervals s in brackets. Standard errors clustered at the hospital-month level. Since the year-month fixed effect was added to the model, the coefficient of $\;{\text{Post}}_{t}$ was not estimated separately. Similarly, the inclusion of hospital fixed effects precluded separate estimation of the coefficient for tertiary_hospital. The ICD-10 refers to the International Classification of Diseases, 10th Revision. “Yes” indicates that the corresponding covariates were included in the regression model. The estimated values of the covariates are reported in the Appendix Tables E1, E2.

## 4 Discussion

In 2020, the study city t shifted the payment model for all locally enrolled BRBMI patients from FFS payments to DRG-PBPS. Using patient-level data and a DID approach, the study revealed that DRG-PBPS reduced hospitalization costs by 9.7% (*p* < 0.01) and LOS by 6.5% (*p* < 0.05). These effects remained robust after accounting for the COVID-19 pandemic and were not attributable to strategic provider behaviors such as patient selection or premature discharge. The impact of DRG-PBPS is consistent across hospitals of different levels. This provides robust evidence to support the claim that DRG-PBPS reduces healthcare resource use among cerebral infarction patients, which is consistent with the findings of Liao et al. (26).

The observed reductions are consistent with the theoretical foundations of DRG-PBPS. Principal–agent theory posits that case-based reimbursement discourages overprovision by rendering additional services financially disadvantageous. Within the DRG-PBPS framework, three design features account for the efficiency gains. First, the floating point value mechanism ensures that increases in service volume dilute financial returns. Second, regional budget coordination embeds hospitals in a collective risk pool, penalizing overspending by any single institution. Third, competitive benchmarking rewards efficient hospitals and penalizes inefficient ones. Together, these mechanisms generate strong incentives for cost containment, directly reflected in the reductions in hospitalization costs and LOS.

Mechanism analysis indicated that the reduction in hospitalization costs was primarily driven by a decrease in drug and consumable costs. Relative to the non-local group, the local group experienced an 11.9% (*p* < 0.01) reduction in drug and consumable costs. Drugs and consumables, being substitutable goods, can generate savings through product substitution or by procuring lower-cost alternatives, making them particularly sensitive to cost-control measures (39, 74). This outcome aligns with the predictions of principal-agent theory: when hospitals face cost-control pressures, they typically reduce expenditures in areas with high managerial discretion and low quality risks (24, 31). Notably, the stability of service and treatment costs, along with diagnostic costs, suggests that efficiency gains were achieved without compromising core clinical services.

Although the DRG-PBPS may incentivize premature discharge, hospitals did not adopt such practices. Compared with the control group, the reform group exhibited no statistically significant changes in 14-day or 90-day readmission rates, while the 30-day readmission rate declined by approximately 2% (*p* < 0.05). Rather than indicating cost-driven premature discharge, this reduction in 30-day readmissions suggests that discharge practices may have improved, implying that efficiency gains were achieved without compromising care quality (4, 61, 62, 66, 73). These findings are consistent with theoretical expectations. Within the DRG-PBPS framework, competition, regulatory oversight, and reputational considerations constrain premature discharge, compelling hospitals to balance cost containment with the preservation of care quality. From a policy perspective, the results reaffirm that DRG-based payment systems with embedded quality monitoring can stabilize or even enhance care quality (75–77). Collectively, this evidence demonstrates that the DRG-PBPS, when coupled with appropriate oversight, can achieve efficiency gains while maintaining or even enhancing care quality.

The reform did not change the severity profiles of admitted patients. Following DRG-PBPS implementation, the CCI of local BRBMI patients was not significantly different from that of the nonlocal group ( β = − 0.072, *p* > 0.1), indicating that providers did not engage in patient selection. This finding is consistent with evidence from U. S. bundled payment studies on cardiac and joint replacement surgeries, which reported no significant changes in patient characteristics (78–80). It underscores the mitigating effect of adjustment mechanisms within the DRG-PBPS design, such as CMI weighting and the proportion of patients over 65. In addition, all study hospitals were public non-profit institutions, which are subject to stronger institutional accountability and social responsibility, further reducing incentives to engage in strategic admission practices.

In summary, the findings validate the theoretical expectations of DRG-PBPS. The reform reduced costs and LOS through mechanisms predicted by principal–agent theory, with savings concentrated in discretionary inputs such as drugs and consumables. Importantly, no evidence of strategic behaviors—such as selective patient admission or premature discharge—was observed under the DRG-PBPS. These findings demonstrate that the DRG-PBPS design—anchored in floating point values, regional budget coordination, and risk adjustment—can realign provider incentives toward efficiency.

To our knowledge, this is the first quasi-experimental study to use disease-specific inpatient records to evaluate the impact of the DRG-PBPS on healthcare resource utilization. It also examines whether premature discharges or other strategic provider responses emerge. The findings show that, when integrated with global budgeting, DRG-PBPS implementation is feasible. It improves resource efficiency and supports cost containment.

This study has the following limitations. First, the sample was limited to patients with cerebral infarction, which may limit generalizability to other diseases or medical procedures that require different resources and have distinct treatment characteristics. Second, differences in medical infrastructure, policy environments, and resource availability across regions may constrain the external validity of the findings. Third, owing to data limitations, the analysis focuses on short-term effects, whereas long-term impacts remain unclear and require further investigation. Fourth, the data are derived from public hospitals, with private hospitals not included in the analysis. Because the response behaviors of private hospitals may differ from those of public institutions, future research should examine this issue. Finally, some behavioral responses, such as coding upgrades and unnecessary hospital stays, were not assessed.

## 5 Conclusion

This study provides robust evidence that China’s DRG-PBPS reform reduced hospitalization costs and LOS for cerebral infarction patients. The reductions were concentrated in drugs and consumables, while spending on services/treatments and diagnostics remained stable. This suggests that savings came from discretionary inputs rather than cuts to essential care. Moreover, these improvements were achieved without evidence of adverse provider behaviors such as premature discharge or selective patient admission. The DRG-PBPS design—anchored in floating point values, regional budget coordination, and risk adjustment—effectively realigns provider incentives toward cost containment. For developing countries with constrained budgets and limited cost-accounting infrastructure, the DRG-PBPS provides a feasible and adaptable pathway for implementing DRG-based payment reforms in inpatient care.

## Data Availability

The datasets presented in this article are not readily available because the datasets employed in this study are exclusively licensed to the authors associated with this project. For those interested in analyzing the original data, it is necessary to submit separate application. Requests to access the datasets should be directed to Jinghua Zhang, jhuzhang@must.edu.mo.
