# Supplementary material for: Impacts of DRG point-based payment system on healthcare resource utilization and provider behavior: a pilot quasi-experimental study in China
Source: Front Public Health. 2025 Oct 7;13:1678259. doi: 10.3389/fpubh.2025.1678259 (PMC12537663; doi:10.3389/fpubh.2025.1678259)
Supplement: Supplementary file 1 [file Table_1.docx]

Supplementary_Material.docx.

**Appendix**

Appendix A The design of Diagnosis-Related Group Point-Based Payment System

Appendix B Full specifications of placebo tests using the difference-in-differences model.

Appendix C Robustness tests using the Propensity Score Matching-Difference in Differences model

Appendix D Full specifications of robustness tests using the difference-in-differences model with COVID-19 pandemic period data excluded.

Appendix E Full specifications of heterogeneity analysis across hospital levels using the Difference-in-Difference-in-Differences model

**Appendix A The design of Diagnosis-Related Group Point-Based Payment System.**

**Figure A1 The design of** **Diagnosis-Related Group Point-Based Payment System**

**Appendix B Full specifications of placebo tests using the difference-in-differences model.**

**Table B1 Placebo test of the impacts of the DRG-PBPS implementation on hospitalization costs and length of stay**

| Variables | LN (hospitalization costs per case) |  | LN(length of stay) |
| --- | --- | --- | --- |
|  |  |  |  |
|  | (1) |  | (2) |
| ${Treat}_{i}$ | 0.020 |  | 0.029 |
|  | (-0.027,0.067) |  | (0.025,0.084) |
| ${Post}_{t}\times{Treat}_{i}$ | -0.065 |  | 0.004 |
|  | (-0.244,0.114) |  | (0.078,0.086) |
| Age (years) | 0.001 |  | -0.001^**^ |
|  | (-0.001,0.002) |  | (0.003,0.000) |
| Gender(Male=1) | 0.002 |  | -0.014 |
|  | (-0.021,0.024) |  | (0.037,0.009) |
| Marital Status(Married=1) | -0.049^**^ |  | -0.038 |
|  | (-0.097,-0.002) |  | (0.087,0.011) |
| Emergency admission (yes=1) | 0.131^***^ |  | -0.038 |
|  | (0.055,0.208) |  | (0.105,0.028) |
| Comorbidities(Yes=1) | 0.157^***^ |  | 0.069^***^ |
|  | (0.119,0.195) |  | (0.028,0.111) |
| Number of secondary diagnoses | 0.044^***^ |  | 0.032^***^ |
|  | (0.037,0.051) |  | (0.025,0.039) |
| Receiving procedures (Yes = 1) | 0.803^***^ |  | 0.112 |
|  | (0.657,0.949) |  | (0.051,0.274) |
| Receiving complicated procedures (Yes=1) | -0.110 |  | 0.017 |
|  | (-0.261,0.041) |  | (0.162,0.195) |
| First 6 characters of ICD-10 code | Yes |  | Yes |
| Hospital fixed effect | Yes |  | Yes |
| Year-month fixed effect | Yes |  | Yes |
| Observations | 6195 |  | 6195 |
| R^2^ | 0.374 |  | 0.102 |

**Notes:** ***, **, and * indicate statistical significance at the 1%, 5%, and 10% levels, respectively. 95% confidence intervals s in brackets. Standard errors clustered at the hospital-month level. Since the year-month fixed effect was added to the model, the coefficient of${Post}_{t}$ was not estimated separately. The ICD-10 refers to the International Classification of Diseases, 10th Revision. “Yes” indicates that the corresponding covariates were included in the regression model.

**Table B2 Placebo test of the impacts** **of the DRG-PBPS implementation on chronic condition indicators and readmission**

| Variables | Charlson Comorbidity Index | 14-day readmission | 30-day readmission | 90-day readmission |
| --- | --- | --- | --- | --- |
|  | (1) | (2) | (3) | (4) |
| ${Treat}_{i}$ | 0.185^**^ | 0.007 | 0.016 | 0.029^*^ |
|  | (0.036,0.334) | (-0.003,0.018) | (-0.004,0.036) | (-0.004,0.062) |
| ${Post}_{t}\times{Treat}_{i}$ | -0.083 | -0.014 | -0.025 | -0.021 |
|  | (-0.271,0.104) | (-0.031,0.004) | (-0.065,0.015) | (-0.064,0.022) |
| Age (years) | 0.005^***^ | -0.001^**^ | -0.001 | -0.001 |
|  | (0.002,0.008) | (-0.002,-0.001) | (-0.001,0.001) | (-0.001,0.001) |
| Gender  (Male=1) | -0.001 | -0.005 | -0.005 | -0.005 |
|  | (-0.057,0.055) | (-0.012,0.002) | (-0.016,0.006) | (-0.019,0.009) |
| Marital Status  (Married=1) | 0.008 | 0.001 | -0.015 | -0.008 |
|  | (-0.112,0.128) | (-0.009,0.011) | (-0.039,0.009) | (-0.037,0.020) |
| Emergency admission (yes=1) | -0.033 | -0.010 | -0.066^***^ | -0.065^***^ |
|  | (-0.176,0.111) | (-0.025,0.004) | (-0.083,-0.048) | (-0.093,-0.038) |
| Comorbidities  (Yes=1) | - | 0.006 | -0.001 | 0.004 |
|  |  | (-0.005,0.017) | (-0.020,0.018) | (-0.022,0.030) |
| Number of secondary diagnoses | - | 0.001 | 0.005^***^ | 0.009^***^ |
|  |  | (-0.001,0.003) | (0.002,0.008) | (0.005,0.013) |
| Receiving procedures (Yes = 1) | - | -0.019^***^ | 0.016 | -0.071 |
|  |  | (-0.030,-0.008) | (-0.106,0.139) | (-0.207,0.065) |
| Receiving complicated procedures (Yes=1) | - | 0.017 | 0.108 | -0.023 |
|  |  | (-0.005,0.040) | (-0.048,0.265) | (-0.207,0.161) |
| First 6 characters of ICD-10 code | Yes | Yes | Yes | Yes |
| Hospital fixed effect | Yes | Yes | Yes | Yes |
| Year-month fixed effect | Yes | Yes | Yes | Yes |
| Observations | 6195 | 6195 | 6195 | 6195 |
| R^2^ | 0.098 | 0.022 | 0.051 | 0.044 |

**Notes:** ***, **, and * indicate significance levels of 1%, 5%, and 10%, respectively. 95% confidence intervals s in brackets. Column (1) estimates the CCI based on equation (1), excluding comorbidities ,secondary diagnoses receiving procedures and receiving complicated procedures as covariates. Since the year-month fixed effect was added to the model, the coefficient of${Post}_{t}$ was not estimated separately. Standard errors clustered at the hospital-month level. The ICD-10 refers to the International Classification of Diseases, 10th Revision. “Yes” indicates that the listed covariates were included in the model.

**Appendix C Robustness tests using the Propensity Score Matching-Difference in Differences model**

**Table C1 Balance test for Propensity Score Matching (before vs. after matching)**

| Variables | Types of samples | Mean | | %bias | t-test | |
| --- | --- | --- | --- | --- | --- | --- |
|  |  | Treated | Control |  | t | p>\|t\| |
| Age (years) | Unmatched | 70.343 | 67.804 | 22.4 | 11.96 | <0.001 |
|  | Matched | 70.343 | 70.399 | -0.5 | -0.41 | 0.684 |
| Gender(Male=1) | Unmatched | 0.564 | 0.641 | -15.7 | -8.19 | <0.001 |
|  | Matched | 0.564 | 0.585 | -4.3 | -3.33 | 0.001 |
| Marital status(Married=1) | Unmatched | 0.912 | 0.884 | 9.6 | 5.22 | <0.001 |
|  | Matched | 0.912 | 0.927 | -4.8 | -4.14 | <0.001 |
| Emergency admission (yes=1) | Unmatched | 0.071 | 0.155 | -26.8 | -15.58 | <0.001 |
|  | Matched | 0.071 | 0.067 | 1.2 | 1.20 | 0.229 |
| Comorbidities(Yes=1) | Unmatched | 0.185 | 0.254 | -16.7 | -9.05 | <0.001 |
|  | Matched | 0.185 | 0.162 | 5.6 | 4.78 | <0.001 |
| Number of secondary diagnoses | Unmatched | 4.436 | 5.281 | -35.7 | -18.77 | <0.001 |
|  | Matched | 4.436 | 4.483 | -2.0 | -1.54 | 0.124 |
| Receiving procedures (Yes = 1) | Unmatched | 0.031 | 0.069 | -10.34 | -10.34 | <0.001 |
|  | Matched | 0.031 | 0.031 | -0.12 | -0.12 | 0.907 |
| Receiving complicated procedures (Yes=1) | Unmatched | 0.023 | 0.056 | -16.9 | -10.07 | <0.001 |
|  | Matched | 0.023 | 0.025 | -0.6 | -0.58 | 0.560 |

**Table C2 Summary of Covariate Balance for Unmatched and Matched Samples**

| Types of samples | Pseudo R² | LR χ² | p-value of LR χ² | Mean Bias | Med Bias | B | R | %Var |
| --- | --- | --- | --- | --- | --- | --- | --- | --- |
| Unmatched | 0.045 | 761.81 | <0.001 | 20.2 | 17.3 | 52.9 | 0.80 | 50 |
| Matched | 0.002 | 61.58 | <0.001 | 2.4 | 1.6 | 10.1 | 1.07 | 100 |

**Notes:** Pseudo R² is from the propensity score model (logit). The LR **χ²** test remains significant due to the large sample size, but the drastic drop in Pseudo R² to near zero confirms the loss of explanatory power.

**Figure C1 Common support – distribution of units across propensity score intervals**

**Figure C2** Covariate balance plot for Propensity Score Matching

**Table C3 Robustness test of the impacts of the DRG-PBPS implementation on hospitalization costs and length of stay using the Propensity Score Matching-Difference in Differences model**

| Variables | LN (hospitalization costs per case) |  | LN(length of stay) |
| --- | --- | --- | --- |
|  | (1) |  | (2) |
| ${Treat}_{i}$ | 0.051^**^ |  | 0.030 |
|  | (0.005,0.097) |  | (-0.014,0.075) |
| ${Post}_{t}\times{Treat}_{i}$ | -0.096^***^ |  | -0.061^**^ |
|  | (-0.149,-0.042) |  | (-0.111,-0.011) |
| Age (years) | 0.000 |  | -0.001^***^ |
|  | (-0.000,0.001) |  | (-0.002,-0.000) |
| Gender(Male=1) | 0.001 |  | -0.008 |
|  | (-0.014,0.017) |  | (-0.023,0.007) |
| Marital Status(Married=1) | -0.056^***^ |  | -0.059^***^ |
|  | (-0.084,-0.029) |  | (-0.088,-0.030) |
| Emergency admission (yes=1) | 0.102^***^ |  | -0.014 |
|  | (0.065,0.140) |  | (-0.046,0.019) |
| Comorbidities(Yes=1) | 0.109^***^ |  | 0.053^***^ |
|  | (0.086,0.131) |  | (0.032,0.075) |
| Number of secondary diagnoses | 0.047^***^ |  | 0.035^***^ |
|  | (0.042,0.051) |  | (0.031,0.039) |
| Receiving procedures (Yes = 1) | 0.770^***^ |  | 0.139^**^ |
|  | (0.660,0.880) |  | (0.025,0.252) |
| Receiving complicated procedures (Yes=1) | 0.115^*^ |  | 0.016 |
|  | (-0.010,0.240) |  | (-0.112,0.145) |
| First 6 characters of ICD-10 code | Yes |  | Yes |
| Hospital fixed effect | Yes |  | Yes |
| Year-month fixed effect | Yes |  | Yes |
| Observations | 15725 |  | 15725 |
| *R*^2^ | 0.420 |  | 0.138 |

**Notes:** ***, **, and * indicate statistical significance at the 1%, 5%, and 10% levels, respectively. 95% confidence intervals s in brackets. Standard errors clustered at the hospital-month level. Since the year-month fixed effect was added to the model, the coefficient of${Post}_{t}$ was not estimated separately. The ICD-10 refers to the International Classification of Diseases, 10th Revision. “Yes” indicates that the corresponding covariates were included in the regression model.

**Table C4 Robustness test of the Impacts of the DRG-PBPS implementation on Chronic Condition Indicator and readmission using the Propensity Score Matching-Difference in Differences model**

| Variables | Charlson Comorbidity Index | 14-day readmission | 30-day readmission | 90-day readmission |
| --- | --- | --- | --- | --- |
|  | (1) | (2) | (3) | (4) |
| ${Treat}_{i}$ | 0.143^***^ | 0.001 | 0.011 | 0.018 |
|  | (0.043,0.242) | (-0.009,0.010) | (-0.007,0.029) | (-0.004,0.040) |
| ${Post}_{t}\times{Treat}_{i}$ | -0.072 | -0.001 | -0.020^**^ | -0.018 |
|  | (-0.191,0.047) | (-0.011,0.010) | (-0.040,-0.001) | (-0.043,0.007) |
| Age (years) | 0.005^***^ | -0.001 | -0.001^*^ | -0.001 |
|  | (0.003,0.007) | (-0.001,0.001) | (-0.001,0.001) | (-0.001,0.001) |
| Gender(Male=1) | 0.046^**^ | -0.003 | -0.005 | -0.008^*^ |
|  | (0.007,0.085) | (-0.007,0.001) | (-0.012,0.001) | (-0.017,0.001) |
| Marital Status(Married=1) | -0.052 | 0.003 | -0.012^*^ | -0.014 |
|  | (-0.123,0.019) | (-0.004,0.009) | (-0.025,0.001) | (-0.031,0.003) |
| Emergency admission (yes=1) | -0.021 | 0.003 | -0.026^***^ | -0.025^***^ |
|  | (-0.107,0.064) | (-0.004,0.011) | (-0.038,-0.014) | (-0.042,-0.008) |
| Comorbidities(Yes=1) | - | 0.004^*^ | 0.003 | 0.008 |
|  |  | (-0.001,0.009) | (-0.008,0.015) | (-0.003,0.020) |
| Number of secondary diagnoses | - | 0.001 | 0.003^***^ | 0.006^***^ |
|  |  | (-0.001,0.002) | (0.001,0.004) | (0.004,0.008) |
| Receiving procedures (Yes = 1) | - | -0.005 | 0.076^**^ | 0.005 |
|  |  | (-0.020,0.010) | (0.004,0.148) | (-0.042,0.052) |
| Receiving complicated procedures (Yes=1) | - | 0.010 | 0.087^*^ | 0.013 |
|  |  | (-0.009,0.028) | (-0.013,0.187) | (-0.038,0.064) |
| First 6 characters of ICD-10 code | Yes | Yes | Yes | Yes |
| Hospital fixed effect | Yes | Yes | Yes | Yes |
| Year-month fixed effect | Yes | Yes | Yes | Yes |
| Observations | 15725 | 15725 | 15725 | 15725 |
| *R*^2^ | 0.114 | 0.013 | 0.058 | 0.044 |

**Notes:** ***, **, and * indicate significance levels of 1%, 5%, and 10%, respectively. 95% confidence intervals s in brackets. Column (1) estimates the CCI based on equation (1), excluding comorbidities ,secondary diagnoses receiving procedures and receiving complicated procedures as covariates. Since the year-month fixed effect was added to the model, the coefficient of${Post}_{t}$ was not estimated separately. Standard errors clustered at the hospital-month level. The ICD-10 refers to the International Classification of Diseases, 10th Revision. “Yes” indicates that the listed covariates were included in the model.

**Appendix D Full specifications of robustness tests using the difference-in-differences model with COVID-19 pandemic period data excluded.**

**Table D1 Robustness tests of the impacts of the DRG-PBPS implementation on hospitalization costs and length of stay with COVID-19 pandemic period data excluded**

| Variables | LN (hospitalization costs per case) |  | LN(length of stay) |
| --- | --- | --- | --- |
|  | (1) |  | (2) |
| ${Treat}_{i}$ | 0.118^***^ |  | 0.041 |
|  | (0.050,0.185) |  | (-0.016,0.097) |
| ${Post}_{t}\times{Treat}_{i}$ | -0.106^**^ |  | -0.090^**^ |
|  | (-0.198,-0.014) |  | (-0.161,-0.019) |
| Age (years) | -0.001 |  | -0.002^***^ |
|  | (-0.002,0.001) |  | (-0.003,-0.001) |
| Gender(Male=1) | -0.007 |  | -0.019 |
|  | (-0.034,0.020) |  | (-0.046,0.007) |
| Marital Status(Married=1) | -0.066^***^ |  | -0.059^**^ |
|  | (-0.108,-0.024) |  | (-0.105,-0.014) |
| Emergency admission (yes=1) | 0.078^***^ |  | -0.009 |
|  | (0.022,0.134) |  | (-0.061,0.043) |
| Comorbidities(Yes=1) | 0.083^***^ |  | 0.034^*^ |
|  | (0.044,0.122) |  | (-0.002,0.070) |
| Number of secondary diagnoses | 0.054^***^ |  | 0.044^***^ |
|  | (0.047,0.061) |  | (0.037,0.050) |
| Receiving procedures (Yes = 1) | 0.715^***^ |  | 0.128 |
|  | (0.472,0.958) |  | (-0.083,0.339) |
| Receiving complicated procedures (Yes=1) | 0.120 |  | 0.113 |
|  | (-0.145,0.386) |  | (-0.123,0.349) |
| First 6 characters of ICD-10 code | Yes |  | Yes |
| Hospital fixed effect | Yes |  | Yes |
| Year-month fixed effect | Yes |  | Yes |
| Observations | 5602 |  | 5602 |
| *R*^2^ | 0.407 |  | 0.155 |

Notes: ***, **, and * indicate statistical significance at the 1%, 5%, and 10% levels, respectively. 95% confidence intervals s in brackets. Standard errors clustered at the hospital-month level. Since the year-month fixed effect was added to the model, the coefficient of${Post}_{t}$ was not estimated separately.The ICD-10 refers to the International Classification of Diseases, 10th Revision. “Yes” indicates that the corresponding covariates were included in the regression model.

**Table D2 Robustness tests of the impacts of the DRG-PBPS Implementation on Chronic Condition Indicator and readmission with COVID-19 pandemic period data excluded**

| Variables | Charlson Comorbidity Index | 14-day readmission | 30-day readmission | 90-day readmission |
| --- | --- | --- | --- | --- |
|  | (1) | (2) | (3) | (4) |
| ${Treat}_{i}$ | 0.158^**^ | 0.007 | 0.030^**^ | 0.029^*^ |
|  | (0.007,0.308) | (-0.004,0.017) | (0.005,0.055) | (-0.003,0.061) |
| ${Post}_{t}\times{Treat}_{i}$ | -0.044 | -0.006 | -0.033^**^ | -0.021 |
|  | (-0.248,0.160) | (-0.019,0.008) | (-0.063,-0.003) | (-0.061,0.018) |
| Age (years) | 0.004^**^ | -0.001 | -0.001 | 0.001 |
|  | (0.001,0.008) | (-0.001,0.001) | (-0.001,0.001) | (-0.001,0.001) |
| Gender(Male=1) | 0.063^**^ | 0.001 | -0.001 | -0.001 |
|  | (0.002,0.125) | (-0.006,0.008) | (-0.011,0.011) | (-0.015,0.014) |
| Marital Status(Married=1) | -0.076 | 0.004 | 0.003 | 0.013 |
|  | (-0.201,0.049) | (-0.003,0.011) | (-0.016,0.021) | (-0.010,0.036) |
| Emergency admission (yes=1) | 0.001 | 0.002 | -0.033^***^ | -0.026^**^ |
|  | (-0.140,0.141) | (-0.007,0.012) | (-0.053,-0.014) | (-0.050,-0.001) |
| Comorbidities(Yes=1) | - | 0.004 | -0.001 | 0.005 |
|  |  | (-0.004,0.012) | (-0.019,0.018) | (-0.014,0.024) |
| Number of secondary diagnoses | - | 0.001 | 0.002 | 0.005^**^ |
|  |  | (-0.001,0.002) | (-0.001,0.005) | (0.001,0.009) |
| Receiving procedures (Yes = 1) | - | -0.011^***^ | 0.193^***^ | 0.090 |
|  |  | (-0.018,-0.004) | (0.067,0.319) | (-0.027,0.206) |
| Receiving complicated procedures (Yes=1) | - | 0.011 | 0.119 | -0.085 |
|  |  | (-0.006,0.028) | (-0.087,0.326) | (-0.199,0.028) |
| First 6 characters of ICD-10 code | Yes | Yes | Yes | Yes |
| Hospital fixed effect | Yes | Yes | Yes | Yes |
| Year-month fixed effect | Yes | Yes | Yes | Yes |
| Observations | 5602 | 5602 | 5602 | 5602 |
| *R*^2^ | 0.131 | 0.015 | 0.043 | 0.037 |

**Notes:** ***, **, and * indicate significance levels of 1%, 5%, and 10%, respectively. 95% confidence intervals s in brackets. Column (1) estimates the CCI based on equation 3, excluding comorbidities , secondary diagnoses receiving procedures and receiving complicated procedures as covariates. Since the year-month fixed effect was added to the model, the coefficient of${Post}_{t}$ was not estimated separately. The ICD-10 refers to the International Classification of Diseases, 10th Revision. “Yes” indicates that the listed covariates were included in the model. Standard errors clustered at the hospital-month level.

**Appendix E Full specifications of heterogeneity analysis across hospital levels using the Difference-in-Difference-in-Differences model**

**Table E1** **Heterogeneity analysis across hospital levels of the impacts of the DRG-PBPS implementation on hospitalization costs and length of stay using the Difference-in-Difference-in-Differences model**

| Variables | LN (hospitalization costs per case) |  | LN(length of stay) |
| --- | --- | --- | --- |
|  | (1) |  | (2) |
| ${Treat}_{i}$ | 0.075^**^ |  | 0.057^*^ |
|  | (0.014,0.137) |  | (-0.001,0.115) |
| ${Post}_{t}\times{Treat}_{i}$ | -0.107^***^ |  | -0.085^**^ |
|  | (-0.180,-0.033) |  | (-0.150,-0.019) |
| ${Post}_{t}\times$*Tertiary_hospital* | 0.075 |  | 0.051 |
|  | (-0.027,0.176) |  | (-0.041,0.144) |
| ${Treat}_{i}\times$*Tertiary_hospital* | -0.065 |  | -0.067 |
|  | (-0.159,0.029) |  | (-0.156,0.023) |
| ${Post}_{t}\times{Treat}_{i}\times$*Tertiary_hospital* | 0.059 |  | 0.079 |
|  | (-0.049,0.167) |  | (-0.022,0.180) |
| Age (years) | 0.001 |  | -0.001^**^ |
|  | (-0.001,0.001) |  | (-0.002,-0.001) |
| Gender(Male=1) | 0.001 |  | -0.008 |
|  | (-0.014,0.017) |  | (-0.023,0.007) |
| Marital Status(Married=1) | -0.056^***^ |  | -0.059^***^ |
|  | (-0.083,-0.029) |  | (-0.087,-0.030) |
| Emergency admission (yes=1) | 0.104^***^ |  | -0.013 |
|  | (0.066,0.141) |  | (-0.046,0.019) |
| Comorbidities(Yes=1) | 0.108^***^ |  | 0.054^***^ |
|  | (0.086,0.131) |  | (0.032,0.075) |
| Number of secondary diagnoses | 0.047^***^ |  | 0.035^***^ |
|  | (0.042,0.051) |  | (0.031,0.039) |
| Receiving procedures (Yes = 1) | 0.759^***^ |  | 0.129^**^ |
|  | (0.649,0.870) |  | (0.015,0.243) |
| Receiving complicated procedures (Yes=1) | 0.121^*^ |  | 0.022 |
|  | (-0.004,0.246) |  | (-0.106,0.151) |
| First 6 characters of ICD-10 code | Yes |  | Yes |
| Hospital fixed effect | Yes |  | Yes |
| Year-month fixed effect | Yes |  | Yes |
| Observations | 15744 |  | 15744 |
| *R*^2^ | 0.423 |  | 0.140 |

Notes: ***, **, and * indicate statistical significance at the 1%, 5%, and 10% levels, respectively. 95% confidence intervals s in brackets. Standard errors clustered at the hospital-month level. Since the year-month fixed effect was added to the model, the coefficient of${Post}_{t}$ was not estimated separately. Similarly, the inclusion of hospital fixed effects precluded separate estimation of the coefficient for tertiary_hospital. The ICD-10 refers to the International Classification of Diseases, 10th Revision. “Yes” indicates that the corresponding covariates were included in the regression model.

**Table E2 Heterogeneity analysis across hospital levels of the impacts of the DRG-PBPS Implementation on Chronic Condition Indicator and readmission using the Difference-in-Difference-in-Differences model**

| Variables | Charlson Comorbidity Index | 14-day readmission | 30-day readmission | 90-day readmission |
| --- | --- | --- | --- | --- |
|  | (1) | (2) | (3) | (4) |
| ${Treat}_{i}$ | -0.031 | 0.004 | 0.010 | 0.020 |
|  | (-0.149,0.087) | (-0.006,0.015) | (-0.013,0.032) | (-0.009,0.048) |
| ${Post}_{t}\times{Treat}_{i}$ | 0.071 | -0.009 | -0.017 | -0.018 |
|  | (-0.075,0.217) | (-0.022,0.003) | (-0.043,0.009) | (-0.053,0.017) |
| ${Post}_{t}\times$*Tertiary_hospital* | 0.453^***^ | -0.012 | 0.016 | 0.016 |
|  | (0.246,0.661) | (-0.032,0.007) | (-0.020,0.052) | (-0.030,0.062) |
| ${Treat}_{i}\times$*Tertiary_hospital* | 0.322^***^ | -0.009 | 0.001 | -0.008 |
|  | (0.135,0.508) | (-0.028,0.010) | (-0.036,0.039) | (-0.054,0.038) |
| ${Post}_{t}\times{Treat}_{i}\times$*Tertiary_hospital* | -0.221^*^ | 0.018 | -0.002 | 0.008 |
|  | (-0.447,0.005) | (-0.004,0.040) | (-0.044,0.039) | (-0.044,0.061) |
| Age (years) | 0.005^***^ | -0.001 | -0.000 | -0.001 |
|  | (0.003,0.007) | (-0.001,0.001) | (-0.001,0.000) | (-0.001,0.001) |
| Gender(Male=1) | 0.047^**^ | -0.003 | -0.005 | -0.008^*^ |
|  | (0.008,0.086) | (-0.007,0.001) | (-0.011,0.002) | (-0.017,0.001) |
| Marital Status(Married=1) | -0.044 | 0.003 | -0.013^*^ | -0.015^*^ |
|  | (-0.116,0.027) | (-0.003,0.009) | (-0.026,0.000) | (-0.032,0.002) |
| Emergency admission (yes=1) | -0.014 | 0.003 | -0.026^***^ | -0.026^***^ |
|  | (-0.100,0.072) | (-0.004,0.010) | (-0.038,-0.014) | (-0.043,-0.009) |
| Comorbidities(Yes=1) | - | 0.005^*^ | 0.004 | 0.009 |
|  |  | (-0.001,0.010) | (-0.008,0.015) | (-0.003,0.020) |
| Number of secondary diagnoses | - | 0.001 | 0.003^***^ | 0.006^***^ |
|  |  | (-0.001,0.002) | (0.001,0.004) | (0.004,0.008) |
| Receiving procedures (Yes = 1) | - | -0.005 | 0.077^**^ | 0.003 |
|  |  | (-0.020,0.010) | (0.005,0.148) | (-0.044,0.050) |
| Receiving complicated procedures (Yes=1) | - | 0.010 | 0.091^*^ | 0.014 |
|  |  | (-0.009,0.028) | (-0.009,0.191) | (-0.037,0.064) |
| First 6 characters of ICD-10 code | Yes | Yes | Yes | Yes |
| Hospital fixed effect | Yes | Yes | Yes | Yes |
| Year-month fixed effect | Yes | Yes | Yes | Yes |
| Observations | 15744 | 15744 | 15744 | 15744 |
| *R*^2^ | 0.117 | 0.013 | 0.059 | 0.044 |

Notes: ***, **, and * indicate statistical significance at the 1%, 5%, and 10% levels, respectively. 95% confidence intervals s in brackets. Standard errors clustered at the hospital-month level. Since the year-month fixed effect was added to the model, the coefficient of${Post}_{t}$ was not estimated separately. Similarly, the inclusion of hospital fixed effects precluded separate estimation of the coefficient for tertiary_hospital. The ICD-10 refers to the International Classification of Diseases, 10th Revision. “Yes” indicates that the corresponding covariates were included in the regression model.
